# Oxidized phospholipid damage signals as modulators of immunity

**DOI:** 10.1098/rsob.240391

**Published:** 2025-07-30

**Authors:** Joon H. Choi, Jonathan C. Kagan

**Affiliations:** ^1^ Harvard Medical School and Division of Gastroenterology, Boston Children’s Hospital, Boston, MA, USA

**Keywords:** innate immunity, oxidized lipids, hyperactive dendritic cells, inflammasomes, PGPC, vaccine adjuvant

## Abstract

Damage-associated molecular patterns (DAMPs) are self-derived molecules released during tissue damage that influence immune responses. Phospholipids, essential to cell membranes and lung surfactants, become oxidized under conditions of cellular stress, forming oxidized phospholipids. Unlike their unoxidized counterparts, oxidized phospholipids function as DAMPs and engage pattern recognition receptors (PRRs) on innate immune cells, activating signalling pathways that regulate immune responses. This activity alters innate immune cells, which in turn modulate the adaptive immune response, ultimately contributing to the pathogenesis of disease. Traditionally considered pro-inflammatory, recent studies reveal a more nuanced role for these lipids, with their effects on immune cells being context dependent. This review examines the mechanisms behind the generation of oxidized phospholipids and their induction in disease. We focus on recent studies that clarify how these lipids affect innate immune cells, leading to downstream effects on adaptive immunity, as well as their direct influence on adaptive immune cells. Finally, we explore therapeutic strategies targeting oxidized phospholipids to regulate immunity, offering insights into their broader role in immune regulation and potential applications in disease prevention.

**Figure:**
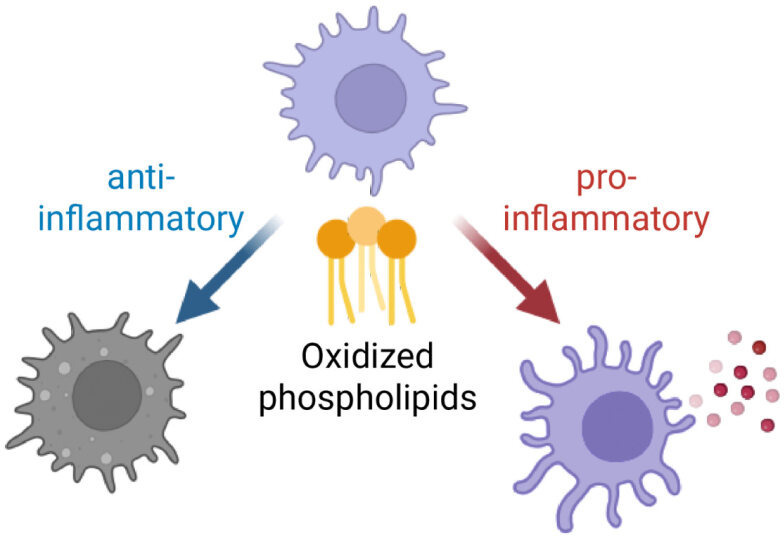

## 1. Introduction

The innate immune system discriminates non-self from self-entities and initiates an immune response to the former to protect our body from potential harm [1]. It serves this purpose by identifying specific molecular patterns produced by microorganisms, which are either absent or found in low abundance within self under conditions of homeostasis. Mammalian cells encode receptors dedicated to sensing these patterns, termed pattern recognition receptors (PRRs) [2]. There are several classes of PRRs, including toll-like receptors (TLRs), retinoic acid-inducible gene I (RIG-I)-like receptors (RLRs) and nucleotide oligomerization domain (NOD)-like receptors (NLRs), among others [3]. Each class of PRRs has distinct ligand specificity and subcellular localization, enabling this network of receptors to detect a wide range of potential pathogens and cellular stress signals. When PRRs are engaged, the responding cells become activated to induce inflammation and adaptive immunity, providing long-term protection [4].

PRRs are best known for their abilities to become activated upon binding conserved molecular patterns found on microbes, known as pathogen-associated molecular patterns (PAMPs). Examples of PAMPs include bacterial lipopolysaccharide (LPS) and peptidoglycan, which are different from the molecules found in the host. Adding another layer of complexity to the immune system, certain biological stress conditions evoke the induction of self-encoded molecules that engage and activate PRRs [2]. These host-derived molecules are referred to as damage-associated molecular patterns (DAMPs) as they are released passively upon cell death or actively secreted in response to cellular stress or injury [5]. Just as PAMPs indicate the presence of a microbial encounter, the detection of DAMPs signals tissue or cellular damage [6]. This detection prompts the recruitment of immune cells to the site of injury, aiding in the resolution of the damage. However, chronic or excessive release of DAMPs can lead to pathological inflammation that exacerbates tissue injury [6]. Understanding the importance of DAMPs: how they are induced, activate inflammation, and are regulated is a focal point of contemporary immunological research. Among the various types of DAMPs, oxidized phospholipids have received attention due to their role in inflammation and disease [7].

The use of oxygen during cellular activities generates reactive oxygen species (ROS). ROS are tightly regulated, with low levels being utilized in physiological processes such as cell proliferation, differentiation and metabolism. However, various stress conditions can disrupt this balance, leading to ROS accumulation and oxidative damage to cellular membranes, proteins and nucleic acids [8]. As an abundant component of cell membranes, phospholipids are primary targets of oxidation [9]. Oxidative modification transforms these phospholipids into signalling molecules that act as DAMPs. Notably, oxPAPC, the oxidation product of palmitoyl-2-arachidonoyl-sn-glycero3-phosphocholine (PAPC), is an example.

In this review, we will discuss the role of oxidized phospholipid DAMPs in the immune system. Early research on oxidized phospholipids has presented conflicting perspectives on whether they are primarily anti-inflammatory or pro-inflammatory, leading to confusion about their role in immunity. However, recent studies have provided a framework for understanding the function of these DAMPs, revealing that the nature of oxidized phospholipids is context dependent and can influence various aspects of immunology. We will provide an overview of how oxidized phospholipids are induced in diseases and their impact on the innate and adaptive immune systems. Lastly, we discuss potential strategies for modulating oxidized phospholipids to prevent and resolve inflammatory disease pathogenesis.

## 2. Diverse means of oxidized phospholipid generation

The oxidation of phospholipids occurs through either enzymatic or non-enzymatic pathways. Enzymatically oxidized phospholipids are generated by lipoxygenases and cyclooxygenases through a highly controlled process, as reviewed recently [10]. In contrast, non-enzymatic oxidation occurs spontaneously due to ROS from various sources, resulting in a broad spectrum of oxidized phospholipid species with potentially distinct biological activities and pathological implications [7]. Lipids from non-enzymatic processes are generally considered more immunostimulatory and damaging than the more regulated enzymatically generated oxidation products [11]. While there are overlaps in the structure and functionality between these lipids, this review will focus on non-enzymatically formed oxidized phospholipids.

**Figure 1.**
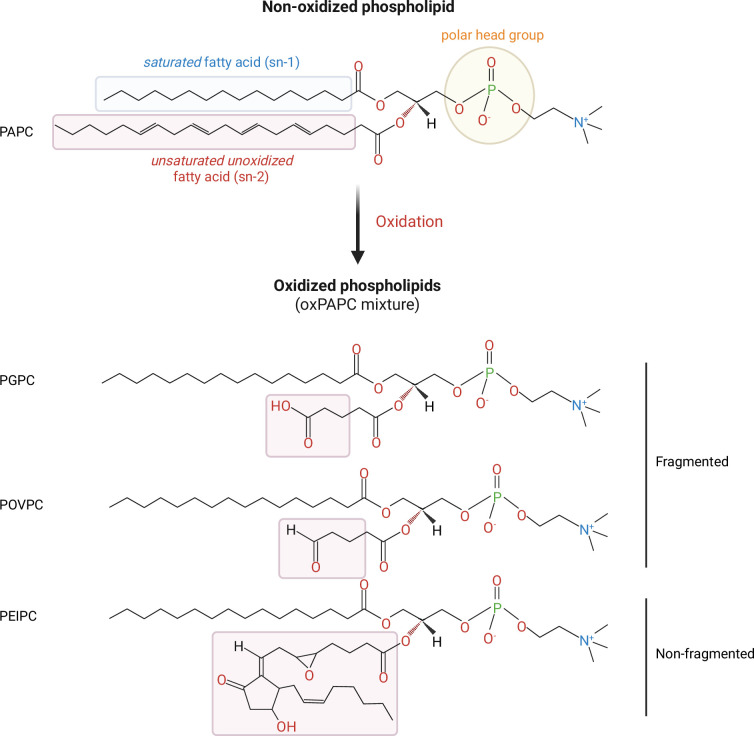
Generation of fragmented and non-fragmented oxidized phospholipids. PAPC contains polyunsaturated fatty acids in the sn-2 position that are susceptible to oxidation. Oxidation of PAPC results in a heterogeneous mixture of oxidized lipid species termed oxPAPC. Oxidation of the polyunsaturated fatty acid chain may lead to oxidative fragmentation, generating short residues in the sn-2 position containing hydroxyl or carbonyl groups. Intramolecular cyclization, rearrangement, and further oxidation produce non-fragmented, full-length oxPAPCs that retain their fatty acid chain. Representative structures of fragmented (PGPC, POVPC) and non-fragmented (PEIPC) oxidized phospholipid species in oxPAPC are depicted. Image created with BioRender.com.

Phospholipids are amphipathic molecules composed of a hydrophilic head and one or more hydrophobic tails derived from fatty acids. Phospholipids that contain polyunsaturated fatty acids, which contain more than one unsaturated double bond in their carbon chain, are more susceptible to oxidation than saturated lipids. In phosphatidylcholines (PCs) such as PAPC, oxidation occurs generally at the sn2 position, the second carbon of the glycerol backbone, that leads to the formation of complex oxidized phospholipid mixtures that are collectively known as oxPAPC (figure 1) [7]. The oxidation of phospholipids occurs in a series of steps [10]. The reaction begins with forming an alkyl radical from free radicals attacking the methylene bridge between double bonds. This alkyl radical then reacts with oxygen to form a peroxyl radical, attacking other polyunsaturated fatty acids and perpetuating the chain reaction. The reaction terminates when two radicals combine to form a non-radical product or when antioxidants neutralize the radicals.

OxPAPC is a heterogeneous mixture of oxidized lipids composed of fragmented or full-length derivatives [12] (figure 1). Fragmented oxPAPCs are created when the polyunsaturated fatty acid chains in phospholipids undergo oxidative breakdown, resulting in shorter acyl chains with reactive functional groups [7,13]. Fragmented oxPAPC products include 1-palmitoyl-2-glutaroyl-sn-glycero-3-phosphocholine (PGPC) and 1-palmitoyl-2-(5-oxovaleroyl)-sn-glycero-3-phosphocholine (POVPC), which contain aldehyde, keto or hydroxy groups at the sn-2 position due to the fragmentation of the original fatty acid chain (figure 1). Non-fragmented full-length oxPAPCs retain their fatty acid chains but undergo structural modifications by adding oxygen-containing functional groups (figure 1). This process forms complex oxidized species such as 1-palmitoyl-2- (5,6-epoxyisoprostane E_2_)-sn-glycero-3-phosphatidylcholine (PEIPC) through intramolecular cyclization and epoxidation, forming cyclopentenone and isoprostane structures [14]. These oxidative modifications of phospholipids are amplified during inflammatory responses, where immune cells produce ROS that drive lipid peroxidation.

Inflammatory responses in innate immune cells, such as macrophages and neutrophils, increase the production of ROS at the site of inflammation [15–18]. This process is mainly achieved through respiratory bursts and cellular enzymes that generate free radicals. The primary sources of ROS in cells include mitochondria, nicotinamide adenine dinucleotide phosphate (NADPH) oxidase and myeloperoxidase [8,18]. Mitochondria produce ROS as by-products of respiration, with the electron transport chain generating superoxide anions, which can lead to lipid peroxidation [8]. NADPH oxidase transfers electrons from NADPH to oxygen, resulting in superoxide radical production, a process particularly active during the respiratory burst in phagocytic cells [18]. Neutrophil cytosolic factor 1 (NCF1) is a crucial component of this enzyme complex, essential for activating NADPH oxidase and promoting ROS production. NCF1 deficiency is associated with reduced ROS production and subsequent lipid peroxidation, thereby alleviating acute lung injury during respiratory infections in mice [9]. Myeloperoxidase, most abundantly expressed in neutrophils, catalyses the production of hypochlorous acid (HOCl) from hydrogen peroxide (H_2_O_2_) and chloride ions (Cl⁻) during the respiratory burst [17]. HOCl is a potent oxidizing agent that can react with phospholipids [19], significantly contributing to oxidative stress and tissue damage during inflammatory responses. Additionally, exogenous sources such as ultraviolet irradiation, smoking and atmospheric ozone can also stimulate the generation of oxidized phospholipids [20]. Oxidative damage occurs when the accumulation of ROS exceeds the cell’s antioxidant capacity, resulting in the modification of various cellular components.

## 3. Induction of oxidized phospholipids in disease

Phospholipids, as fundamental structural components of cellular membranes across diverse organs, are universally susceptible to oxidative damage under conditions of cellular stress [7]. Consequently, oxidized phospholipids have been identified in a wide range of pathological settings, from the lungs of patients with highly contagious respiratory infections [9] to the coronary arteries of individuals with chronic cardiovascular disease [21]. Their widespread occurrence across tissues and disease states highlights their potential importance as mediators of inflammation and tissue damage. Therefore, understanding the induction and diverse roles of these lipids across different pathological contexts is essential for understanding their broader immunological impact.

Phospholipids are particularly abundant in lung surfactants [22]. They make up to 80% of the total mass of lung surfactants, with PCs constituting up to 85% of the phospholipids [23]. The high metabolic activity and continual exposure to microbes and pollutants lead to the generation of ROS, making the lungs especially vulnerable to oxidative stress [22]. For instance, lung bronchoalveolar lavage (BAL) fluid from individuals infected with respiratory viruses can show a significant increase in ROS levels up to 70-fold [24]. Consequently, phospholipids in the lungs are susceptible to oxidation.

While early studies suggested that lung cells infected with influenza viruses can produce cellular oxidized phospholipids *in vitro* [25], subsequent research verified the induction of oxidized phospholipids in human and mouse lungs following highly pathogenic viral infections [9]. The formation of oxidized phospholipids was mainly due to the production of ROS in pulmonary phagocytic cells induced upon infection in the lungs. Generation of oxidized phospholipids was identified at lung injury sites, including the BAL fluid and lung tissues of mice and patients infected with H5N1 avian influenza virus and the 2003 severe acute respiratory syndrome (SARS) coronavirus. The oxidized phospholipids induce the pro-inflammatory cytokine IL-6 production and acute lung injury by engaging the PRR TLR4 on alveolar macrophages [9]. Multiple studies have confirmed the accumulation of diverse oxidized phospholipids in the lungs upon respiratory viral infection, highlighting the broad relevance of oxidized phospholipid induction upon viral infections [26–29].

The formation of oxidized phospholipids has also been observed in several bacterial infections, particularly those that affect the lungs [9,30]. For instance, oxidized phospholipid induction was observed in *Bacillus anthracis*-infected rhesus monkeys and rabbits, monkeypox-infected cynomolgus monkeys, and *Yersinia pestis*-infected cynomolgus monkeys that developed lung plague [9]. These observations indicate that bacterial infections causing severe lung pathology can similarly induce the production of oxidized phospholipids. The induction of oxidized phospholipids has also been reported in the case of leprosy caused by *Mycobacterium leprae* [30]. In human leprosy lesions, PEIPC was found to accumulate, particularly in macrophage-dense areas. PEIPC specifically inhibited the upregulation of the antigen presentation molecule, CD1b, on dendritic cells (DCs), preventing effective antigen presentation and potentially hindering the activation of *M. leprae*-reactive immune responses [30].

The underlying mechanism for the induction of oxidized phospholipids in the aforementioned viral and bacterial infections could be a response to PAMPs induced during infection. Indeed, oxidized phospholipids are produced in humans and mice upon acute stimulation of PRRs [31]. LPS or polyinosinic:polycytidylic acid (poly(I:C))-treated mice exhibit induction of oxPAPC in the blood and the spleen. Among immune cells, macrophages showed the most significant accumulation of these lipids. Blockade of oxidized phospholipids in mice reduced inflammation and prevented death, indicating that oxidized phospholipids amplify inflammatory response, contributing to mortality in severe infections. Mechanistically, oxidized phospholipids suppress levels of the anti-inflammatory cytokine IL-10 by inhibiting Akt signalling [31]. These findings highlight how microbial infections can potentially drive the accumulation of oxidized phospholipids through PAMPs and PRR interactions. Similarly, the role of oxidized phospholipids extends beyond infections, with their presence also identified in the tumour microenvironment (TME) [32].

The TME can be immunosuppressive due to metabolic factors that differ from those in normal tissue [32–34]. These metabolic changes present in the TME include hypoxia, nutrient deprivation and increased lactic acid, which suppress anti-tumour immunity [35–38]. Recent studies have shown that the TME also accumulates higher levels of oxidized phospholipids, which interfere with immune cell activity and promote cancer cell metastasis [32,39–41]. Lipid profiling of the TME from mice with melanoma B16 and colorectal MC38-implanted tumours revealed elevated levels of oxidized phospholipids in both tumours compared to normal skin [32]. This was associated with increased oxidized low-density lipoprotein (oxLDL) uptake and lipid peroxidation in CD8+ TILs relative to splenocytes [32]. Further work revealed that tumour-infiltrating CD8^+^ T cells exposed to oxidized phospholipid-containing oxLDL become dysfunctional, resulting in reduced production of tumour necrosis factor-alpha (TNFα) and interferon-gamma (IFNγ) [32]. Similarly, increased levels of oxidized phospholipids, such as POVPC and PGPC, were found in malignant breast tumour tissue samples compared to adjacent normal tissue [40]. These lipids induced tumour cell metastasis by promoting epithelial–mesenchymal transition (EMT) and autophagy [40]. These studies independently validate the presence of oxidized phospholipids in the TME and indicate their role in promoting the immunosuppressive environment, either by impairing CD8+ T cells or enhancing the metastatic potential of cancer cells.

Beyond their involvement in infections and cancer, oxidized phospholipids also have a substantial impact on non-communicable, lifestyle-related diseases such as atherosclerosis, aortic stenosis and hepatic steatosis [42]. Recent studies have examined the impact of oxidized phospholipids in these conditions, using transgenic mice that can specifically neutralize oxidized phospholipid, oxPAPC [43–45]. These studies show that these transgenic mice, with reduced levels of oxPAPC, exhibit slower progression of atherosclerosis [43] and decreased hepatic inflammation and fibrosis [44,45]. Additionally, there was a noticeable decrease in inflammatory markers and lower expression of pro-inflammatory cytokines [43], suggesting that oxidized phospholipids serve as disease markers and contribute to pathology.

As such, oxidized phospholipids are induced during various disease processes and play a crucial role in pathogenesis, acting as DAMPs. To better understand their impact on immune responses, it is essential to explore how these lipids are detected and recognized by innate immune sensors within the cell, particularly through PRRs.

## 4. Innate immune detection of oxidized phospholipids

Before discussing how oxidized phospholipids influence innate and adaptive immune cells, here we focus on the cellular PRRs reported to recognize and detect oxidized phospholipids DAMPs. Soluble PRRs have been reviewed elsewhere [7].

TLRs are a class of PRRs that detect a wide variety of PAMPs and DAMPs. Depending on the type of TLR and the specific ligand it recognizes, they are located on the cell surface or within endosomes [3]. Once the PRRs are engaged, TLRs dimerize and coordinate with adapter proteins such as MyD88 and TRIF, forming supramolecular organizing centres (SMOCs) that facilitate the initiation and amplification of downstream signalling cascades [46]. This assembly activates transcription factors, including NF-κB, AP-1 and IRFs. Among the various TLRs, TLR4 is particularly significant in mediating immune responses to oxidized phospholipids [9]. In alveolar macrophages, oxPAPC triggers inflammatory responses through the TLR4-TRIF-TRAF6 pathway, leading to the induction of interleukin 6 (IL-6) [9]. Genetic studies also reveal TLR2 as a sensor of oxPAPC, in which oxPAPC-mediated inflammatory responses are reduced in TLR2-deficient mice and macrophages [47].

CD14 is a glycosylphosphatidylinositol-anchored protein functioning primarily as a co-receptor for TLR4 [48,49]. In addition to LPS, CD14 serves as a receptor for oxidized phospholipids [50]. Oxidized phospholipid-CD14 complexes are internalized through endocytosis, delivering the lipids to intracellular compartments where they can activate the NLRP3 inflammasome by a mechanism that remains unclear. CD14’s role in transporting oxidized phospholipids also affects subsequent cellular responses to LPS in which oxPAPC binding to CD14 can prevent the interaction of LPS with CD14, thereby inhibiting TLR4-mediated responses to LPS [50]. This competitive mechanism displays the regulatory function of CD14 in balancing pro-inflammatory and anti-inflammatory signals within the immune system.

Caspase-4 in humans and its murine counterpart, caspase-11, are involved in the non-canonical inflammasome pathway [51,52], particularly in response to intracellular LPS and oxidized phospholipids [52,53]. However, the specific interaction and downstream activation outcome of LPS and oxidized phospholipids are distinctly different. While oxPAPC binds directly to the catalytic domain of caspase-11, promoting its oligomerization but not its enzymatic activity, LPS binds to the caspase activation and recruitment domain (CARD), leading to its oligomerization and activation [53]. When LPS engages caspase-11, it triggers the non-canonical inflammasome pathway and results in pyroptotic cell death, resulting in a transient burst of IL-1β production [51]. In contrast, oxPAPC-induced caspase-11 oligomerization is sufficient to induce NLRP3 inflammasome assembly and IL-1β secretion through the pore-forming protein gasdermin D without causing pyroptosis [50,53,54]. This state of activation, termed hyperactivation, allows myeloid cells to remain viable while secreting IL-1β [52].

Scavenger receptors (SRs) are a diverse family of PRRs involved in the recognition and internalization of oxidized phospholipids and lipid-associated proteins [55–58]. Class A scavenger receptors (SR-As) primarily recognize oxLDL and very-low-density lipoprotein (VLDL), promoting foam cell formation [55,57]. While oxidized phospholipids can be generated during oxidation of these lipoproteins, studies suggest the primary binding target of SR-As is the modified apolipoprotein B, rather than the oxidized phospholipid components themselves [57]. In contrast, Class B scavenger receptors (SR-Bs), including SR-B1 (SCARB1) and CD36 (SR-B3, fatty acid translocase), directly recognize oxidized phospholipid species [56,59]. This interaction is driven by recognition of the terminal negatively charged carboxylate at the sn-2 position of the phospholipid [59]. CD36, in particular, is a versatile receptor expressed on phagocytes, which can bind various ligands, including long-chain fatty acids and oxidized phospholipids [7,60,61]. CD36 has a hydrophobic pocket that accommodates the fatty acyl chains of oxidized phospholipids, facilitating their capture and internalization [62,63]. In macrophages, CD36 is crucial for the uptake of oxLDL, leading to the formation of foam cells and contributing to atherosclerosis [55,64–68]. Upon binding, CD36 activates multiple signalling pathways, primarily by recruiting Src family kinases and activating NF-κB leading to the production of proinflammatory cytokines such as TNFα and IL-1β [67,69,70]. Additionally, CD36 engagement with oxidized phospholipids can trigger signalling pathways that induce oxidative stress responses, including the upregulation of NADPH oxidase activity and the production of ROS, thus perpetuating inflammation and oxidative damage [63,70,71].

Through recognition by PRRs, oxidized phospholipids trigger signalling pathways that are central to the activation and modulation of innate immune cells. These receptors detect oxidized phospholipids as markers of cellular damage and initiate inflammatory responses that drive immune activity. This interaction significantly affects the functional roles of innate immune cells, shaping their contributions to inflammation and immunity.

## 5. Influence of oxidized phospholipids on innate immune cell functions

**Figure 2.**
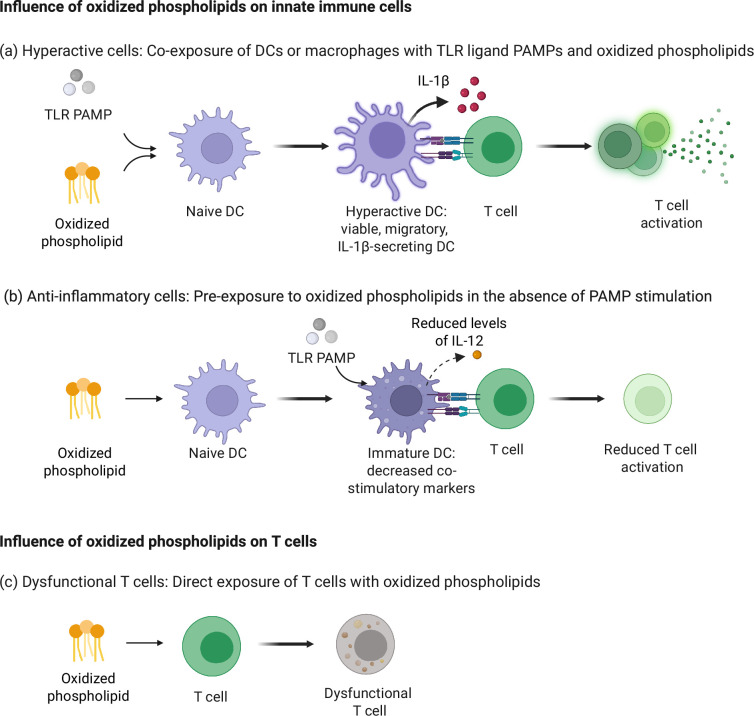
Influence of oxidized phospholipids on innate and adaptive immune cells. Examples of the influence of oxidized phospholipids on immune cells are depicted, focusing on their impact on dendritic cells (DCs) and T cells. (a) Upon co-exposure to TLR ligands and oxidized phospholipids, DCs become hyperactive, secreting IL-1β while maintaining viability. Hyperactive DCs display enhanced migration to lymph nodes and interact with T cells to induce effective T-cell activation. (b) Pre-treatment of DCs with oxidized phospholipids, in the absence of TLR PAMPs, reduces DC activation upon subsequent TLR stimulation. This results in decreased co-stimulatory marker expression, reduced IL-12 production and ultimately diminished T-cell activation. (c) Direct exposure of T cells to oxidized phospholipids increases lipid uptake, causing T cells to become dysfunctional and exhausted, with reduced cytotoxic effector function. Image created with BioRender.com.

DCs are central to the adaptive immune response, uniquely capable of orchestrating the generation of effective CD4^+^ and CD8^+^ T-cell responses [72]. Five key activities of DCs are necessary to initiate durable T-cell responses [73]. These activities include the presentation of antigens through the major histocompatibility complex (MHC), expression of T-cell co-stimulatory molecules (e.g. CD40, CD80 and CD86), DC migration to lymph nodes, the effector T-cell activating cytokine (e.g. type I interferon, IL−12) production and the production of IL-1β. Among the various cytokines that DCs secrete, IL-1β plays a crucial role in T-cell differentiation and memory formation, and its release is influenced by the molecular stimuli that DCs encounter [74–76]. When DCs are exposed to non-infectious microbes, the PAMPs detected lead to the production of several cytokines. However, PAMP-exposed DCs are unable to secrete IL-1β, as they cannot activate the inflammasomes required for pro-IL-1β cleavage and release [77]. However, upon encountering pathogens, DCs typically release IL-1β through pyroptotic cell death [78]. Pyroptosed DCs, being dead and inert, cannot interact with naive T cells, which is essential for T-cell differentiation. Recent studies have identified a distinct state of DC activation known as hyperactivation, which is associated with the release of IL-1β that is dependent on the inflammasome but does not involve pyroptotic cell death [52,53,79]. The transition to hyperactivity occurs when DCs are exposed to both TLR ligands and oxPAPC, or oxPAPC components such as PGPC or POVPC [50,53,79] (figure 2a).

The sustained release of IL-1β from living (i.e. hyperactive) DCs allows for continued interaction with T cells in the lymph nodes, thereby enhancing T-cell activation [79,80]. Hyperactive DCs also exhibit increased expression of the costimulatory marker CD40 and production of IL-12-p70, as compared to their active and pyroptotic counterparts, further influencing the activation of T-cell responses [80]. A recent study expands our understanding of the immunological impact of hyperactive stimulation, showing that macrophages and DCs activated with LPS and PGPC uniquely induce highly branched, F-actin-rich filopodia, in a greater degree than those found on pyroptotic corpses, which persist on these activated cells [81]. These structures function as novel alarm signals recognized by the C-type lectin receptor CLEC9A (DNGR1) on incoming DCs, triggering SYK phosphorylation and activating DC antigen presentation pathways that upregulate costimulatory markers, promoting T-cell activation and the initiation of adaptive immunity [81].

As one of the key activities of DCs required to activate T cells, DC migration to lymph nodes is tightly regulated by the C–C chemokine receptor type 7 (CCR7) receptor and its ligands, the chemokines (C–C motif) ligand 19 (CCL19) and CCL21 [82,83]. CCR7 detects gradients of these chemokines in the lymphatics, guiding DCs towards lymph nodes, where they present antigen fragments to naive T cells [84]. Upon exposure to the oxidized phospholipid PGPC, hyperactive DCs display a marked induction of CCR7, correlating with enhanced DC migration to the lymph nodes in mice [79]. Transcriptomic analysis reveals the upregulation of migration-associated genes, such as Rhob, Rhoc and Rac1, in hyperactive DCs, in contrast to their active and pyroptotic counterparts [79]. Notably, hyperactive DCs retained their enhanced migratory capacity even in aged mice [80]. This finding is particularly significant, as ageing is often associated with a decline in immune function, including reduced DC migration to lymph nodes [85–87]. The ability of hyperactive DCs to overcome this age-related defect underscores their potential to enhance DC-mediated immune responses in the elderly.

However, the immunomodulatory effects of oxidized phospholipids are context dependent. When DCs encounter oxidized phospholipids without microbial molecules, mimicking sterile tissue injury, they adopt anti-inflammatory characteristics (figure 2b). DCs pre-treated with oxPAPC show reduced activation upon later exposure to PAMPs [14,88,89]. This pre-treatment inhibits TLR3 and TLR4-mediated activation and pro-inflammatory cytokine IL-12 production by blocking NF-κB activation, dependent on the redox-sensitive transcription factor Nrf2 [89]. Additionally, different oxidized phospholipids within oxPAPC have varied effects on DCs. For instance, while PGPC and POVPC showed no effect, PEIPC decreases CD1 expression on DCs, impacting their lipid antigen-presenting capabilities to T cells [30].

While DCs and macrophages are both professional phagocytes involved in the innate immune response, macrophages are versatile, capable of switching between pro-inflammatory and anti-inflammatory tissue repair roles, allowing them to both initiate and resolve inflammation [90,91]. Their interaction with various lipid-based metabolites is crucial in regulating this transition between inflammatory phenotypes [92]. Similar to DCs, oxidized phospholipids are potent inducers of hyperactivity in macrophages, promoting the release of inflammasome-mediated IL-1β without triggering pyroptosis [50,54] (figure 2a). This activity is underpinned by the metabolic rewiring of macrophages, specifically induced by oxPAPC [93]. While macrophages stimulated with LPS rely primarily on anaerobic glycolysis, oxPAPC enhances mitochondrial activity by boosting both oxidative phosphorylation and aerobic glycolysis [93]. This hypermetabolic state leads to elevated levels of key metabolites such as acetyl-CoA and oxaloacetate. In turn, oxaloacetate increases hypoxia-inducible factor 1-alpha levels, which supports the enhanced production of IL-1β. Specific components of oxPAPC, such as PEIPC, further amplify this hypermetabolic and hyperactivated state in macrophages [93].

Studies highlighting the pro-inflammatory activities of oxidized phospholipids in macrophages further support these inflammatory shifts. For instance, alveolar macrophages exposed to oxPAPC have been shown to exacerbate inflammation in the lungs by producing IL-6 [9]. Similarly, other studies demonstrate that the recognition of oxPAPC by macrophages promotes a pro-inflammatory state characterized by the production of IL-1β and TNFα [43,44]. These findings collectively emphasize the critical role of oxidized phospholipids in driving macrophage-mediated inflammatory responses.

While relatively short exposure to oxidized phospholipids triggers a robust pro-inflammatory response, prolonged exposure can induce an anti-inflammatory state in macrophages. Studies suggest that chronic exposure to oxidized phospholipids induces specific changes in the gene expression profile of macrophages, resulting in their polarization to an anti-inflammatory phenotype [14,89]. Among these gene changes, a set regulated by Nrf2 was observed in macrophages [14]. Nrf2 activation by oxidized phospholipids stimulates the transcription of genes involved in reducing oxidative damage and promoting cell survival [14].

Foam cells are commonly found in atherosclerosis lesions and fibrosis, where they contribute to plaque formation and the progression of atherosclerosis [65,67,94]. Foam cell formation in macrophages is primarily driven by the uptake of oxidized phospholipids via SRs, such as CD36, resulting in the accumulation of lipid droplets within the cells [65,67]. These lipid-laden macrophages transform into foam cells, which then secrete cytokines and chemokines, attracting additional immune cells to the lesion site. Notably, research on mice targeted for the neutralization of oxPAPC displayed reduced foam cell formation and macrophage lipid uptake, thereby mitigating fibrosis and inflammation in liver steatohepatitis [45,95].

Complementing the orchestrated actions of DCs and macrophages in immune regulation, neutrophils act as immediate responders to infection, utilizing distinct mechanisms to rapidly neutralize pathogens and control inflammation [96]. Neutrophils are among the first immune cells recruited to sites of infection and defend against pathogens through mechanisms such as oxidative burst and the formation of neutrophil extracellular traps (NETs). The oxidative burst rapidly produces ROS to kill pathogens, while NETs help neutralize them [97].

Neutrophils’ oxidative burst is a potent antimicrobial system that requires tight regulation to prevent tissue damage from the sudden release of ROS [98–100]. Studies indicate that the degree of phospholipid oxidation influences the oxidative burst in neutrophils [101]. When incubated with unoxidized phospholipid PAPC, neutrophils enhance ROS production. In contrast, oxidized phospholipids, including oxPAPC, inhibit ROS production by preventing the assembly of the NADPH oxidase complex, a key player in the oxidative burst. This inhibition specifically targets ROS production without impacting the viability of neutrophils or their other cellular activities [101]. This may act as a balancing mechanism, allowing lipid oxidation to modulate the neutrophil immune response to pathogens while minimizing tissue damage.

Recent findings have revealed that oxidized phospholipids also influence neutrophil NET formation [102]. NETs are web-like structures composed of decondensed chromatin and granular content released by neutrophils during a cell death process called NETosis [103,104]. The study found that oxidized phospholipids are essential for NETosis, influencing the signalling events that lead to chromatin decondensation and nuclear swelling, both critical for effective NET formation [102]. In the absence of lipid peroxidation, these structural changes necessary for NET formation do not occur.

Beyond their influence on individual innate immune cells, oxidized phospholipids can also modulate innate immune function more broadly by altering membrane architecture, particularly through the regulation of lipid raft dynamics. Lipid rafts are cholesterol- and sphingolipid-rich microdomains within the plasma membrane that serve as essential platforms for the clustering of protein receptors and the regulation of signal transduction [105,106]. These structures are therefore integral for both innate and adaptive immune signalling [107]. Studies find that oxidized phospholipids can intercalate into lipid rafts and subsequently alter their behaviour through distinct biophysical mechanisms that vary depending on the molecular structure of the inserted lipid species [107–110]. Hydroperoxidized lipids, POPCOOH, promote raft formation and increase the size of rafts by inducing hydrophobic mismatch, while fragmented oxidized lipids such as POVPC disrupt raft integrity and increase membrane permeability [108,110]. These structural modifications directly impact the localization and activation of PRRs such as TLRs, thereby influencing downstream inflammatory signalling pathways.

While oxPAPC components, in combination with TLR agonists, induce a hyperactivated state that can prevent pyroptotic cell death, excessive lipid peroxidation has been associated with a distinct form of cell death known as ferroptosis. Unlike other regulated forms of cell death, ferroptosis is uniquely defined by the accumulation of iron-dependent lipid peroxidation, which serves as both a marker and a mediator of this process [111,112]. Lipidomic studies reveal significantly higher levels of oxidized phospholipids, primarily oxidized phosphatidylethanolamine (oxPE) and phosphatidylserine (oxPS), during ferroptosis compared to apoptosis, necroptosis or pyroptosis [113]. The core mechanism underlying ferroptosis involves accumulation of these lipid species due to impaired activity of glutathione peroxidase 4 (GPX4), an essential enzyme responsible for neutralizing phospholipid hydroperoxides [114,115]. A recent study identified phospholipids containing two polyunsaturated fatty acyl tails (PC-PUFA2s) as direct inducers of ferroptosis [116]. PC-PUFA2s interact with the mitochondrial electron transport chain, inducing mitochondrial ROS production, with the primary site of peroxidation being the endoplasmic reticulum [116]. While lipids containing these acyl chains rarely occur and are not present in oxPAPC, several *in vitro* studies suggest that specific oxPAPC components can induce ferroptosis in certain cell types. PGPC and POVPC have been reported to trigger ferroptosis by suppressing GPX4 expression, in endothelial and vascular smooth muscle cells, respectively [117,118]. PGPC impairs endothelial function by increasing intracellular ferrous iron levels, thereby promoting ferroptosis through enhanced lipid peroxidation [117]. POVPC, like PGPC, promotes ferroptosis in vascular smooth muscle cells, contributing to vascular calcification [118]. These findings further highlight how oxidized phospholipids can disrupt cellular homeostasis and alter cell fate.

Oxidized phospholipids modulate the activity of various innate immune cells, shaping the initial immune response and ultimately exerting profound downstream effects on adaptive immunity. The innate immune system’s ability to recognize and respond to PAMPs and oxidized phospholipids directly influences the activation and direction of adaptive immunity. We will next discuss how oxidized phospholipids impact T-cell activity, a central aspect of the adaptive immune response.

## 6. Oxidized phospholipids’ influence on T-cell immunity

While the innate immune system provides a rapid, non-specific first line of defence, adaptive immunity offers a delayed but highly specific, long-term protection [4,119]. T cells are central to adaptive immune system, with the primary subsets being CD8^+^ and CD4^+^ T cells. CD8^+^ T cells are responsible for directly killing infected and cancerous cells, recognizing antigens presented by MHC molecules on the surface of these cells [120]. CD4^+^ T cells are further subdivided into several subsets, including T helper 1 (Th1), Th2, Th17, T follicular helper (Tfh) and regulatory T (Treg) cells, each with distinct roles and cytokine profiles [121,122]. The activation of T cells is a complex process that necessitates extensive interaction and communication with innate immune cells, particularly DCs [73]. Here we will discuss the influence of oxidized phospholipid-modified DCs on T-cell activation as well as the modulation of T-cell activities upon direct interaction with oxidized phospholipids.

Hyperactive DCs, stimulated by TLR agonists and oxidized phospholipids, have been explored in the context of anti-tumour immunity for their ability to enhance T-cell activation [79,80]. Unlike TLR-activated DCs, hyperactive DCs enhance T-cell differentiation through their increased lymph node-homing capacity and sustained secretion of IL-1β [79] (figure 2a). In young mice, this leads to a significant proliferation of antigen-specific effector CD8^+^ T cells and heightened IFN-γ production [79,80]. In contrast, aged populations typically exhibit a decline in naive CD8^+^ T cells and impaired DC migration, leading to reduced responses to cancer immunotherapy [85,87,123]. Hyperactive DCs, however, correct these deficiencies by inducing a Th1-skewed response, with CD4^+^ T cells primarily producing IFN-γ and minimizing Th2 cytokine induction [80]. This results in robust anti-tumour responses across both age groups: in young mice, strong CD8^+^ T-cell cytotoxicity facilitates tumour clearance, whereas in aged mice, the response shifts to cytolytic CD4^+^ helper T-cell activity, directly killing tumour cells and enhancing the effectiveness of immunotherapy [80].

Unlike hyperactive DCs, naive DCs pre-exposed to oxPAPC show decreased costimulatory marker expression and reduced IL-12 production in response to subsequent exposure to PAMPs [14,88,89] (figure 2b). The inhibitory effects of oxPAPC on DC activation led to a diminished ability of DCs to stimulate T cells, resulting in lower T-cell proliferation and suppression of Th1 differentiation and cytokine production [14,88,89]. Similarly, among oxPAPC constituents, PECPC containing epoxycyclopentenone exhibits anti-inflammatory function in myeloid cells, contributing to a decrease in Th1 responses [14]. These studies highlight the role of oxidized phospholipids in modulating DC activity and, subsequently, influencing downstream T-cell responses. Beyond their influence on innate immune cells, oxidized phospholipids have also been shown to directly impact T cells, with recent studies elucidating these effects on T-cell functionality [32,39,124] (figure 2c).

The TME, atherosclerotic plaques and sites of inflammation, which contain high levels of oxidized phospholipids, increase the likelihood of T cells directly encountering these lipids [32,41]. To mimic these conditions, human peripheral blood T cells were activated *in vitro* with anti-CD3 and CD28 and subsequently exposed to oxPAPC. The study found that oxPAPC inhibited the proliferation of activated T cells, particularly through PGPC but not POVPC [39]. Even upon restimulation, there was no proliferation, indicating T-cell anergy, a state of functional unresponsiveness [39,125]. In the presence of oxPAPC, activated T cells displayed reduced expression of activation markers CD25, CD63 and CD69, along with lower levels of classical Th1 cytokines, including IL-2 and IFN-γ, suggesting a direct inhibitory effect of oxidized phospholipids on T-cell responses [39] (figure 2c).

Further research has examined the influence of lipid metabolites on T cells within the TME, revealing elevated levels of oxidized phospholipids in various tumours [32]. CD8^+^ T cells in the TME take up surrounding lipids via the SR CD36, leading to increased lipid peroxidation and activation of the p38 mitogen-activated protein kinase (MAPK) pathway [32]. This metabolic shift in CD8+ tumour-infiltrating lymphocytes impairs cytotoxic anti-tumour activity and effector function, reflected by increased surface expression of the immune checkpoint receptors PD-1 and TIM-3, classical markers of T-cell exhaustion (figure 2c). In contrast, CD36-deficient CD8^+^ T cells exhibit lower lipid peroxidation levels and, consequently, improved cytotoxic cytokine production capabilities, demonstrating that oxidized phospholipid uptake through CD36 promotes the exhaustion of T cells [32].

Another study focusing on mouse regulatory T-cell subsets found that direct treatment with oxPAPC reduces the overall viability of Tregs [124]. This leads to a reduced population of cells exhibiting a Th1 phenotype, which relies on IFN-γ signalling. When Tregs treated with oxPAPC were transferred into hyperlipidaemic low-density lipoprotein receptor (LDLr) knockout mice, they lost their protective ability to prevent the progression of atherosclerosis, suggesting that oxPAPC impairs the function and differentiation of Tregs [124] (figure 2c).

So far, we have discussed the prevalence of oxidized phospholipids in various diseases and their impact on both innate and adaptive immune responses. Collectively, these studies illustrate how oxidized phospholipid DAMPs modulate immune dynamics and contribute to disease progression. In the final section, we will explore potential strategies for targeting oxidized phospholipids, considering their application as vaccine adjuvants or therapeutic targets.

## 7. Therapeutic applications of oxidized phospholipids in immune modulation

The COVID-19 pandemic has underscored the importance of vaccines and the need for more effective vaccine strategies [126]. This is especially critical for the elderly as ageing leads to gradual dysregulation of immune functions, resulting in reduced vaccine efficacy [127]. However, numerous studies indicate that the usage of effective adjuvants can rejuvenate immune responses to vaccines in the aged population [127,128].

The potential of oxidized phospholipids as vaccine adjuvants lies in their ability to induce hyperactive DCs, leading to a more durable and effective T-cell response. Immunizing mice with DC hyperactivating agents such as LPS and PGPC have been shown to serve as an effective adjuvant to tumour antigens in both therapeutic and prophylactic settings, effectively protecting against various tumours by inducing robust CD8^+^ T-cell responses [79]. Notably, hyperactive DCs can enhance anti-tumour immunity in aged mice by overcoming DC migratory defects and boosting CD4^+^ T-cell activity [80]. This suggests that harnessing hyperactive DCs in vaccine strategies could improve the efficacy of tumour vaccines, particularly in older populations.

The application of oxidized phospholipids as a DC vaccine strategy raises several pertinent questions: which other diseases might benefit from this approach, and what additional T-cell subsets could hyperactive DCs modulate? Beyond cancer immunotherapy, this method could be investigated for infectious diseases, where a robust and sustained T-cell response is essential for targeting evolving variants [52]. Hyperactive DCs may also stimulate other T-cell subsets, such as regulatory T cells and helper T cells, to modulate immune responses across diverse therapeutic settings.

While the *ex vivo* application of oxidized phospholipids can enhance immune responses by hyperactivating DCs, neutralizing endogenously induced oxidized phospholipids presents an alternative therapeutic strategy that could potentially impede disease progression. Several studies have incorporated the natural antibody E06 IgM to support this approach [43–45]. An early investigation identified robust auto-antibody titres against oxLDL in atherosclerosis-prone apolipoprotein E knockout mice [43,129]. Splenic hybridoma B-cell lines from these mice led to the discovery of the monoclonal IgM antibody E06, which specifically binds to the PC head group of oxidized phospholipids and not to free fatty acids. Since its discovery, the E06 antibody has been used to detect these lipids and their association with various diseases, including atherosclerosis, lung injury and fibrosis [9,32,43]. A subsequent study generated mice constitutively expressing the single-chain variable fragment of E06 in hypercholesterolemic Ldlr knockout mice, which substantially reduced atherosclerosis [43] and diet-induced hepatic steatosis [44]. Similar findings were observed using adeno-associated virus (AAV)-mediated hepatic expression of E06, which prevented the progression of hepatic fibrosis [45]. Mechanistically, the introduction of E06 prevented the uptake of oxidized phospholipids and oxLDL in macrophages and hepatocytes, resulting in reduced inflammatory responses and attenuated production of TNFα and IL-1β [44,45].

These findings provide the foundation for therapeutic strategies that neutralize endogenous oxidized phospholipids, potentially reducing inflammation and slowing disease progression, particularly in conditions associated with chronic or excessive expression of these lipids. It remains important to explore whether neutralizing oxidized phospholipids would be beneficial in acute inflammatory conditions such as sepsis or highly pathogenic viral infections. Additionally, it has been observed that the E06 antibody efficiently blocks a subset of lipid species in the oxPAPC mixture, not all [43]. This underscores the significance of studying the immune-modulatory function of individual lipids to understand their role in disease development and improve the effectiveness of future therapeutic strategies targeting oxidized phospholipids.

## 8. Conclusion

The induction of oxidized phospholipids has been observed in a range of diseases, including microbial infections, cancer and chronic inflammatory conditions. These lipids influence both the innate and adaptive arms of immunity, underscoring their role in immune modulation and disease pathogenesis. Their context-dependent behaviour, amplifying inflammation in some settings while suppressing it in others, demonstrates the complexity of their interactions with immune cells. This dual capacity highlights the need for a deeper understanding of the pathways that drive the induction of endogenous oxidized phospholipids and their impact on immunity. How do individual lipid species elicit distinct effects on immune cells? What are the interactions between specific lipid species and PRRs? What regulatory factors control oxidized phospholipid levels across different diseases? Additionally, understanding how oxidized phospholipid-modified immunity influences tissue-specific host responses, including tissue damage and recovery, will provide insights into disease progression. With this knowledge, strategies to regulate oxidized phospholipid levels can be refined to either enhance immune responses or prevent excessive inflammation, opening new avenues for improving immunotherapy.

## Data Availability

This article has no additional data.
